# Bound-state energy spectrum and thermochemical functions of the deformed Schiöberg oscillator

**DOI:** 10.1038/s41598-023-47235-0

**Published:** 2023-11-21

**Authors:** A. D. Ahmed, E. S. Eyube, E. Omugbe, C. A. Onate, P. Timtere

**Affiliations:** 1 Department of Physics, Faculty of Physical Sciences, Modibbo Adama University, P.M.B. 2076, Yola, Adamawa State Nigeria; 2 Department of Physics, University of Agriculture and Environmental Sciences, P.M.B. 1038, Umuagwo, Imo State Nigeria; 3 Department of Physics, Kogi State University, Anyigba, Nigeria

**Keywords:** Chemistry, Physics

## Abstract

In this study, a diatomic molecule interacting potential such as the deformed Schiöberg oscillator (DSO) have been applied to diatomic systems. By solving the Schrödinger equation with the DSO, analytical equations for energy eigenvalues, molar entropy, molar enthalpy, molar Gibbs free energy and constant pressure molar heat capacity are obtained. The obtained equations were used to analyze the physical properties of diatomic molecules. With the aid of the DSO, the percentage average absolute deviation (PAAD) of computed data from the experimental data of the ^7^Li_2_ (2 ^3^Π_g_), NaBr (X ^1^Σ^+^), KBr (X ^1^Σ^+^) and KRb (B ^1^Π) molecules are 1.3319%, 0.2108%, 0.2359% and 0.8841%, respectively. The PAAD values obtained by employing the equations of molar entropy, scaled molar enthalpy, scaled molar Gibbs free energy and isobaric molar heat capacity are 1.2919%, 1.5639%, 1.5957% and 2.4041%, respectively, from the experimental data of the KBr (X ^1^Σ^+^) molecule. The results for the potential energies, bound-state energy spectra, and thermodynamic functions are in good agreement with the literature on diatomic molecules.

## Introduction

The relevance of wave functions in quantum mechanics is the adequate information they provide about the quantum mechanical system being studied, hence the growing need for accurate numerical and approximate analytical bound state solutions of the non-relativistic and relativistic wave equations for a given potential energy function. The potential energy function is the medium through which a quantum mechanical system interacts with particles and molecules in its neighborhood. Examples of interaction potentials include the Tietz potential, Schiöberg potential, Hua potential, Rosen-Morse potential and Woods-Saxon potential^1–5^.

A diatomic molecule oscillator is a potential energy function used to describe the rotational-vibrational states of a molecular system. To qualify as a diatomic molecule oscillator, a potential energy function must satisfy some prescribed conditions also known as the Varshni conditions^6^. Oscillators are modeled using molecular spectroscopic parameters such as the vibrational–rotational coupling coefficient (*α*_e_), equilibrium harmonic vibrational frequency (*ω*_e_), equilibrium bond length (*r*_e_), and equilibrium dissociation energy (*D*_e_). These parameters are usually determined experimentally or by ab initio calculations.

The solutions of the Schrödinger equation (SE) have been obtained analytically with different potential energy models. Expressions for the bound-state energies have been successfully used to investigate the thermodynamic, optical, magnetic and other physical properties of substances^7–21^. Some of the techniques used to obtain the bound state solutions of the wave equations are the asymptotic iteration method^22^, quantization rules^23,24^, the supersymmetric quantum mechanics approach^25^, the Nikiforov-Uvarov method and its parametric versions^26,27^, and the Laplace transformation method^28^.

Thermodynamic functions have immense applications in science and technology. For instance, enthalpy and entropy measurements have been used to determine the melting points of organic molecules, and to detect diseases in plants^29,30^. The thermoplastic property, transition and melting points of nanostructures have also been investigated through measurements of heat capacity^31,32^. In a very recent advancement, the Gibbs free energy equation developed from the well-known Fu-Wang-Jia (FWJ) oscillator has been used to establish the equilibrium constant for the water gas shift reaction^33^.

The solution canonical partition function is a prelude to obtaining statistical-mechanical models (or analytical equations) for the calculation of thermo-chemical properties of gaseous molecules. The partition function takes into account; the vibrational, rotational and translational effects of the diatomic system. Analytical equations for the prediction of the molar entropy (S), enthalpy (H), Gibbs free energy (H), and isobaric heat capacity (C_*p*_) exist in the literature, some examples can be found in Refs.^34–58^. Different potential functions have been employed in the literature to construct analytical model equations^6,59–66^.

The present study is centered on the Schiöberg potential energy function. Previously, the bound-state solutions of the Schrödinger and Dirac equations have been obtained with the Schiöberg potential^67,68^. Using the Varshni conditions^6^, Wang and coworkers demonstrated the equivalence of the Manning-Rosen, Deng-Fan and Schiöberg potentials^64^. The Schiöberg oscillator incorporates three independent input parameters viz *D*_e_, *r*_e_ and *ω*_e_.

In the quest to model an efficient version of the Schiöberg oscillator, the authors in Ref.^69^ employed the transformation *r* → *r*−*r*_0_ and the Varshni conditions^6^ to construct the reparameterized Schiöberg potential. The reparameterized Schiöberg oscillator is expected to encapsulate four independent input parameters: *D*_e_, *r*_e_, *ω*_e_ and *α*_e_, nevertheless, the explicit form of the parameter *r*_0_ was not deduced. Many diatomic molecule oscillators have been used by physicists and chemists to predict the thermochemical properties of gaseous molecules^6,55,59,61–65^. However, for this purpose, the deformed Schiöberg potential has not been considered in the literature. It must be emphasized that q-deforming a potential energy function and subsequently subjecting it to the Varshni conditions for a diatomic molecule potential yields an equivalent model to the reparameterized version^35^. For this reason, the present study primary objectives are to obtain the energy spectra and thermochemical functions of the deformed Schiöberg oscillator. The remaining parts of the paper is organized as follows: In section “Construction of the DSO”, the deformed Schiöberg oscillator is constructed. In section “Equation for the energy spectra of the DSO”, explicit equation for the energy spectra is derived. Thermochemical functions are obtained in section “Thermochemical functions of the DSO”. The results of numerical calculations are presented in section “Results and discussion”. A brief conclusion of the work is given in section “Conclusions”.

## Construction of the DSO

In this section, the deformed Schiöberg oscillator (DSO) is constructed by employing the Varshni conditions^6^. The suggested model potential is given by

1 $$ {\text{U }}\left( r \right) \, = {\text{ U}}_{0} \left\{ {{1 }{-}\sigma {\text{coth}}_{q} \left( {\alpha r} \right)} \right\}^{{2}} , $$

where, coth_*q*_ (*αr*) = cosh_*q*_ (*αr*)/sinh_*q*_ (*αr*), cosh_*q*_ (*αr*) = ½ (e^*αr*^ + e^−*αr*^), sinh_*q*_ (*αr*) = ½ (e^*αr*^−e^−*αr*^), *r* is the interparticle separation, U_0_ is the depth of the potential well, *q*, *α* and *σ* are potential parameters. Evidently, (1) is the q-deformed version given in Ref.^64^. The main difference between Eq. (1) and expression (1) of Ref.^70^ lies in the functional forms of the two models.

Equation (1) is a diatomic molecule oscillator if it satisfies the following conditions^6^

2 $$ {\text{U}}\prime \, \left( r \right) \, = \, 0, {\text{U }}\left( {r \to \, \infty } \right) \, {-}{\text{ U }}\left( {r \to r_{{\text{e}}} } \right) \, = D_{{\text{e}}} ,\;\;{\text{U}}\prime \prime \, \left( {r_{{\text{e}}} } \right) \, = M_{0} \left( {{2}\pi c\omega_{{\text{e}}} } \right)^{{2}} , $$

where the prime in (2) denotes the derivative with respect to *r*, the speed of light is designated *c*, and *μ* is the reduced mass of a molecule. Inserting Eq. (1) into each of the expressions in (2) gives

3 $$ q = \, \left( {{1 }{-}\alpha \varepsilon } \right){\text{ e}}^{{{2}\gamma }} , $$

where *σ* = tanh_*q*_* γ*, U_0_ = *D*_e_ (1−*σ*)^−2^, $\varepsilon = \tfrac{1}{{{\uppi }c\omega_{\text{e}} }}\left( {\tfrac{{2D_{\text{e}} }}{{M_{0} }}} \right)^{{\tfrac{1}{2}}}$, and *γ* = *αr*_e_. The next step is to determine the potential screening parameter, *α*. The *α*_e_-*ω*_e_ relationship given in publication^64^ can be used, viz

4 $$ \alpha_{\text{e}} = - \frac{{6{\text{B}}_{\text{e}}^{2} }}{{\omega_{\text{e}} }}\left\{ {1 + \frac{{r_{\text{e}} }}{3}\frac{{{{{\rm U}^{\prime\prime\prime}}}\left( {r_{\text{e}} } \right)}}{{{{{\rm U}^{\prime\prime}}}\left( {r_{\text{e}} } \right)}}} \right\}, $$

where B_e_ = *ħ*/4π*cM*_0_*r*_e_^2^, *ħ* = *h*/2π, *h* being the Planck constant. U″(*r*_e_) and U‴(*r*_e_) are obtained from Eq. (1) as

5 $$ {\text{U}}^{\prime \prime} \, \left( {r_{{\text{e}}} } \right) \, = { 2}\alpha^{{2}} {\text{U}}_{0} \left( {{1}/\sigma {-}\sigma } \right)^{{2}} , \;\;\; {{{\rm U}^{\prime\prime\prime} }}\left( {r_{{\text{e}}} } \right) \, = \, {-}{ 12}\alpha^{{3}} {\text{U}}_{0} \left( {{1 }{-}{ 1}/\sigma } \right)^{{2}} /\sigma . $$

Putting Eq. (5) into (4) and simplifying leads to

6 $$ \alpha = - \frac{1}{{2r_{\text{e}} }} + \frac{2}{\varepsilon } - \frac{{\alpha_{\text{e}} \omega_{\text{e}} }}{{12{\text{B}}_{\text{e}}^{2} r_{\text{e}} }}. $$

## Equation for the energy spectra of the DSO

In this section, an analytical equation for the energy spectra is derived by solving the radial SE confined by the DSO. Different analytical methods for solving the SE exist in the literature^22–28^. However, owing to the simplicity of the parametric Nikiforov-Uvarov (PNU) technique^27^, it is considered in this work.

### A brief outline of the PNU method

The PNU method gives that with the aid of a suitable coordinate transformation, a second-order differential equation of the hypergeometric-type can be expressed as^27^

7 $$ {{{\rm u}^{\prime\prime}}}_{n\ell } \left( z \right) + \frac{{\alpha_{1} - \alpha_{2} z}}{{z\left( {1 - \alpha_{3} z} \right)}}{{{\rm u}^{\prime}}}_{n\ell } \left( z \right) + \frac{{ - \tau_{1} z^{2} + \tau_{2} z - \tau_{3} }}{{z^{2} \left( {1 - \alpha_{3} z} \right)^{2} }}{\text{u}}_{n\ell } \left( z \right) = 0, $$

where *α*_*j*_ (*j* = 0, 1, 2) are constant coefficients, *n* = 0, 1, 2, … is the vibrational (or principal) quantum number and *ℓ* = 0, 1, 2, … is the rotational (or orbital momentum) quantum number. The quantization condition leading to energy spectra is written as^27^

8 $$ \left( {\alpha_{2} - \alpha_{3} } \right)n + \alpha_{3} n^{2} - \left( {2n + 1} \right)\alpha_{5} + \left( {2n + 1} \right)\left( {\sqrt {\alpha_{9} } + \alpha_{3} \sqrt {\alpha_{8} } } \right) + \alpha_{7} + 2\alpha_{3} \alpha_{8} + 2\sqrt {\alpha_{8} \alpha_{9} } = 0, $$

where

9 $$ \begin{aligned} \alpha_{4} & = \tfrac{1}{2}\left( {1 - \alpha_{1} } \right),\quad \alpha_{5} = \tfrac{1}{2}\left( {\alpha_{2} - 2\alpha_{3} } \right),\quad \alpha_{6} = \alpha_{5}^{2} + \tau_{1} \\ \alpha_{7} & = 2\alpha_{4} \alpha_{5} - \tau_{2} ,\;\alpha_{8} = \alpha_{4}^{2} + \tau_{3} ,\;\alpha_{9} = \alpha_{3} \alpha_{7} + \alpha_{3}^{2} \alpha_{8} + \alpha_{6} . \\ \end{aligned} $$

### Analytical equation for the energy levels of the DSO by the PNU method

The radial SE for a particle of mass *M*_0_ moving in a radial potential field, U (*r*) is given by

10 $$ \frac{{{\text{d}}^{2} {\text{u}}_{n\ell } \left( r \right)}}{{{\text{d}}r^{2} }} + \frac{{2M_{0} }}{{\hbar^{2} }}\left\{ {E_{n\ell } - {\text{U}}\left( r \right) - \frac{{J\hbar^{2} }}{{2M_{0} r^{2} }}} \right\}{\text{u}}_{n\ell } \left( r \right) = 0, $$

where *J* = *ℓ* (*ℓ* + 1) is the angular momentum of the system, u_*nℓ*_ (*r*) is the radial wave function and *E*_*nℓ*_ is the bound-state energy eigenvalue. Owing to the presence of the factor *r*^-2^ in the centrifugal term, expression (10) has no exact solution with the potential (1), except for the special case where *ℓ* = 0 (the pure vibrational state). Nevertheless, an approximate analytical solution can be obtained with the help of approximation models. For small values of *r*, a Pekeris-type approximation scheme can be written for *r*^-2^ as follows

11 $$ r^{ - 2} \approx d_{1} + d_{2} \coth_{q} \left( {\alpha r} \right) + d_{3} \coth_{q}^{2} \left( {\alpha r} \right), $$

where the constant coefficients *d*_*j*_ (*j* = 1, 2, 3) are deduced by the procedures outlined in Ref.^71^ as

12 $$ \begin{aligned} d_{1} & = \frac{1}{{r_{\text{e}}^{2} }}\left( {1 - \frac{{3\sinh_{{q^{2} }} 2\gamma }}{2\gamma q} + \frac{{3\sinh_{{q^{2} }}^{2} 2\gamma }}{{4\gamma^{2} q^{2} }} - \frac{{\sinh_{{q^{4} }} 4\gamma }}{{4\gamma q^{2} }}} \right) \\ d_{2} & = \frac{{\sinh_{q}^{2} \gamma }}{{r_{\text{e}}^{2} }}\left( {\frac{4}{\gamma q} - \frac{{3\sinh_{{q^{2} }} 2\gamma }}{{\gamma^{2} }} + \frac{{2\cosh_{{q^{2} }} 2\gamma }}{{\gamma q^{2} }}} \right) \\ d_{3} & = \frac{{\sinh_{q}^{2} \gamma }}{{r_{\text{e}}^{2} }}\left( { - \frac{3}{{2\gamma^{2} q}} + \frac{{3\cosh_{{q^{2} }} 2\gamma }}{{2\gamma^{2} q^{2} }} - \frac{{\sinh_{{q^{2} }} 2\gamma }}{{\gamma q^{2} }}} \right) \\ \end{aligned} $$

Inserting Eqs. (1) and (11) into (10) gives

13 $$ \frac{{{\text{d}}^{2} {\text{u}}_{n\ell } \left( r \right)}}{{{\text{d}}r^{2} }} + \left\{ {\frac{{2M_{0} \left( {E_{n\ell } - {\text{U}}_{0} } \right)}}{{\hbar^{2} }} - Jd_{1} + \left( {\frac{{4M_{0} {\text{U}}_{0} \sigma }}{{\hbar^{2} }} - Jd_{2} } \right)\coth_{q} \left( {\alpha r} \right) - \left( {\frac{{2M_{0} {\text{U}}_{0} \sigma^{2} }}{{\hbar^{2} }} + Jd_{3} } \right)\coth_{q}^{2} \left( {\alpha r} \right)} \right\}{\text{u}}_{n\ell } \left( r \right) = 0. $$

Using the substitution *z*^-1^ = 1−*q*^-1^ e^2*αr*^, Eq. (13) is transformed to

14 $$ {{{\rm u}^{\prime\prime}}}_{n\ell } \left( r \right) + \frac{1 - 2z}{{z\left( {1 - z} \right)}}{{{\rm u}^{\prime}}}_{n\ell } \left( r \right) + \frac{{ - \tau_{1} z^{2} + \tau_{2} z - \tau_{3} }}{{z^{2} \left( {1 - z} \right)^{2} }}{\text{u}}_{n\ell } \left( r \right) = 0, $$

where

15 $$ \tau_{1} = \frac{{2M_{0} {\text{U}}_{0} \sigma^{2} }}{{\alpha^{2} \hbar^{2} }} + \frac{{Jd_{3} }}{{\alpha^{2} }},\;\tau_{2} = \frac{{2M_{0} {\text{U}}_{0} \sigma \left( {\sigma - 1} \right)}}{{\alpha^{2} \hbar^{2} }} + \frac{{J\left( {2d_{3} + d_{2} } \right)}}{{2\alpha^{2} }},\;\tau_{3} = \frac{{M_{0} \left( {D_{\text{e}} - E_{n\ell } } \right)}}{{2\alpha^{2} \hbar^{2} }} + \frac{{J\left( {d_{3} + d_{2} + d_{1} } \right)}}{{4\alpha^{2} }}. $$

By comparing expressions (14) and (7), one obtains *α*_1_ = 1, *α*_2_ = 2, *α*_3_ = 1. Using these results in Eq. (9) gives *α*_4_ = *α*_5_ = 0, *α*_6_ = *τ*_1_, *α*_7_ = – *τ*_2_, *α*_8_ = *τ*_3_ and *α*_9_ = *τ*_1_−*τ*_2_ + *τ*_3_. Inserting the values of *α*_2_,* α*_3_,* α*_4_, *α*_5_, *α*_6_, *α*_7_, *α*_8_ and *α*_9_ into (8) yields

16 $$ \tau_{3} = \frac{1}{4}\left\{ {n + \frac{1}{2} \pm \sqrt {\tau_{1} + \frac{1}{4}} - \frac{{\tau_{1} - \tau_{2} }}{{n + \frac{1}{2} \pm \sqrt {\tau_{1} + \frac{1}{4}} }}} \right\}^{2} . $$

Using the expressions in (15) to eliminate *τ*_1_, *τ*_2_ and *τ*_3_ in (16), the expressions for bound-state energy

17 $$ E_{n\ell } = D_{\text{e}} + \frac{{J\left( {d_{3} + d_{2} + d_{1} } \right)\hbar^{2} }}{{2M_{0} }} - \frac{{\alpha^{2} \hbar^{2} }}{{2M_{0} }}\left\{ {n + \frac{1}{2} \pm \sqrt {\frac{{2M_{0} {\text{U}}_{0} \sigma^{2} }}{{\alpha^{2} \hbar^{2} }} + \frac{{Jd_{2} }}{{\alpha^{2} }} + \frac{1}{4}} - \frac{{\frac{{2M_{0} {\text{U}}_{0} \sigma }}{{\alpha^{2} \hbar^{2} }} - \frac{{Jd_{2} }}{{2\alpha^{2} }}}}{{n + \frac{1}{2} \pm \sqrt {\frac{{2M_{0} {\text{U}}_{0} \sigma^{2} }}{{\alpha^{2} \hbar^{2} }} + \frac{{Jd_{2} }}{{\alpha^{2} }} + \frac{1}{4}} }}} \right\}^{2} . $$

The pure vibrational state energy *E*_*n*0_ → *E*_*n*_ is obtained by letting *ℓ* = 0 in Eq. (17) to obtain

18 $$ E_{n} = D_{\text{e}} - \frac{{\alpha^{2} \hbar^{2} }}{{2M_{0} }}\left\{ {n + \frac{1}{2} \pm \sqrt {\frac{{2M_{0} {\text{U}}_{0} \sigma^{2} }}{{\alpha^{2} \hbar^{2} }} + \frac{1}{4}} - \frac{{\frac{{2M_{0} {\text{U}}_{0} \sigma }}{{\alpha^{2} \hbar^{2} }}}}{{n + \frac{1}{2} \pm \sqrt {\frac{{2M_{0} {\text{U}}_{0} \sigma^{2} }}{{\alpha^{2} \hbar^{2} }} + \frac{1}{4}} }}} \right\}^{2} . $$

The maximum vibrational quantum number is deduced from the expression *E*′_*n*_ (*n*_max_) = 0, substituting (18) into this expression gives

19 $$ n_{\max } = \pm \left( {\frac{{2M_{0} {\text{U}}_{0} \sigma }}{{\alpha^{2} \hbar^{2} }}} \right)^{{\tfrac{1}{2}}} - \left\{ {\frac{1}{2} \pm \left( {\frac{{2M_{0} {\text{U}}_{0} \sigma^{2} }}{{\alpha^{2} \hbar^{2} }} + \frac{1}{4}} \right)^{{\tfrac{1}{2}}} } \right\}. $$

*n*_max_ is essentially a positive integer, which is the value of *n* at which the energy of the system is a maximum.

## Thermochemical functions of the DSO

Having obtained the equation for vibrational state energies, in this section, some important analytical models for the prediction of thermochemical properties of substances are developed for the DSO. The canonical partition function from which the thermodynamic expressions are deduced is first derived. The canonical partition function is written as Z (*T*) = Z_vib_Z_rot_Z_tra_, where *T* is the temperature of the system, Z_vib_, Z_rot_, and Z_tra_ are the vibrational, rotational and translational partition functions, respectively^44,55^. The vibrational partition function depends on the oscillator used to model the diatomic system, it is given as^34^

20 $$ {\text{Z}}_{{{\text{vib}}}} = \sum\limits_{n\,\, = \,\,0}^{{n_{\max } }} {{\text{exp}}\left( { - \frac{{E_{n} }}{{{\text{k}}_{{\text{B}}} T}}} \right)} , $$

where *β* = 1/(k_B_*T*), k_B_ is the Boltzmann constant. Putting Eq. (18) into (20) gives

21 $$ {\text{Z}}_{{{\text{vib}}}} = \exp \left( { - \omega } \right)\sum\limits_{n\,\, = \,\,0}^{{n_{\max } }} {\varphi \left( n \right)} , $$

where

22 $$ \omega = \frac{{D_{\text{e}} }}{{{\text{k}}_{B} T}},\quad \varphi \left( n \right) = \exp \left\{ {\varsigma \left( {n + \delta - \frac{\kappa }{n + \delta }} \right)} \right\}^{2} ,\quad \varsigma = \frac{\alpha \hbar }{{\sqrt {2M_{0} {\text{k}}_{{\text{B}}} T} }}. $$

The series in (21) can be evaluated with the help of the modified Poisson summation formula^72^. The modified Poisson summation approach is used here because it is simple to implement and has yielded very accurate results with many oscillator models such as those in Refs.^35,38,73,74^. Other methods for evaluating the vibrational partition function including the phase space sampling method and the Euler-Maclaurin summation approach are given in Refs.^75,76^. Based on the modified Poisson summation formula, one can write^72^

23 $$ \sum\limits_{n\,\, = \,\,0}^{{n_{\max } }} {\varphi \left( n \right)} = \frac{1}{2}\left\{ {\varphi \left( 0 \right) - \varphi \left( {n_{\max } + 1} \right)} \right\} + \sum\limits_{k = \, - \infty }^{k = \,\,\infty } {\int\limits_{0}^{{n_{\max } + 1}} {\varphi \left( y \right){\text{exp}}\left( { - {\text{i}}2{\uppi }ky} \right){\text{d}}y} } . $$

Substituting the second expression in (22) into the right-hand side of (23) and expanding out the summation gives

24 $$ \begin{aligned} \sum\limits_{n\,\, = \,\,0}^{{n_{\max } }} {\varphi \left( n \right)} & = \frac{1}{2}\left\{ {\exp \left( {\lambda_{0}^{2} } \right) - \exp \left( {\lambda_{1}^{2} } \right)} \right\} + \int\limits_{0}^{{n_{\max } + 1}} {\exp \left\{ {\varsigma \left( {y + \delta - \frac{\kappa }{y + \delta }} \right)} \right\}^{2} {\text{d}}y} \\ & \;\;\;\, + \sum\limits_{k = - \infty }^{ - 1} {\int\limits_{0}^{{n_{\max } + 1}} {{\text{exp}}\left\{ {\varsigma^{2} \left( {y + \delta - \frac{\kappa }{y + \delta }} \right)^{2} - {\text{i}}2{\uppi }ky} \right\}{\text{d}}y} } + \sum\limits_{k = 1}^{\infty } {\int\limits_{0}^{{n_{\max } + 1}} {{\text{exp}}\left\{ {\varsigma^{2} \left( {y + \delta - \frac{\kappa }{y + \delta }} \right)^{2} - {\text{i}}2{\uppi }ky} \right\}{\text{d}}y} } . \\ \end{aligned} $$

where $\lambda_{0} = \varsigma \left( {\delta - \tfrac{\kappa }{\delta }} \right)$, $\lambda_{1} = \varsigma \left( {n_{\max } + 1 + \delta - \tfrac{\kappa }{{n_{\max } + 1 + \delta }}} \right)$. The last-two terms in (24) include quantum correction terms. For the moderate to high temperature range of diatomic systems, the quantum correction terms are small and can be ignored. Therefore, expression (24) is recast as

25 $$ \sum\limits_{n\,\, = \,\,0}^{{n_{\max } }} {\varphi \left( n \right)} = \frac{1}{2}\left\{ {\exp \left( {\lambda_{0}^{2} } \right) - \exp \left( {\lambda_{1}^{2} } \right)} \right\} + \int\limits_{0}^{{n_{\max } + 1}} {\exp \left\{ {\varsigma \left( {y + \delta - \frac{\kappa }{y + \delta }} \right)} \right\}^{2} {\text{d}}y} . $$

Using the substitution *z* = *ς* {*y* + *δ*−*κ*/(*y* + *δ*)}, followed by the mapping *x* = (*z*^2^ + 2*ςκ*^2^)^½^ to evaluate the integral, the summation in (25) is obtained as

26 $$ \sum\limits_{n\,\, = \,\,0}^{{n_{\max } }} {\varphi \left( n \right)} = \frac{1}{2}\left\{ {\exp \left( {\lambda_{0}^{2} } \right) - \exp \left( {\lambda_{1}^{2} } \right)} \right\} - \frac{{\sqrt {\uppi } }}{4\varsigma }\left\{ {{\text{Erfi}}\lambda_{0} - {\text{Erfi}}\lambda_{1} + \exp \left( { - 4\kappa \varsigma^{2} } \right)\left( {{\text{Erfi}}\eta_{0} - {\text{Erfi}}\eta_{1} } \right)} \right\}, $$

Thus, inserting (26) into (21), the vibrational partition function is obtained in compact form as

27 $$ {\text{Z}}_{{{\text{vib}}}} = {\text{ A}}_{0} {-}{\text{ A}}_{{1}} {-}{\text{ A}}_{{2}} {-}{\text{ A}}_{{3}} , $$

where

28 $$ \begin{aligned} {\text{A}}_{0} & = \frac{1}{2}\exp \left( {\lambda_{0}^{2} - \omega } \right),\quad {\text{A}}_{1} = \frac{1}{2}\exp \left( {\lambda_{1}^{2} - \omega } \right),\quad {\text{A}}_{2} = \frac{{\sqrt {\uppi } }}{4\varsigma }\exp \left( { - \omega } \right)\left( {{\text{Erfi}}\lambda_{0} - {\text{Erfi}}\lambda_{1} } \right){,} \\ {\text{A}}_{3} & = \frac{{\sqrt {\uppi } }}{4\varsigma }\exp \left( { - 4\kappa \varsigma^{2} - \omega } \right)\left( {{\text{Erfi}}\eta_{0} - {\text{Erfi}}\eta_{1} } \right), \\ \end{aligned} $$

Based on the formalism of the rigid-rotor approximation for diatomic molecules, the rotational and translational components of the partition function are expressed as^36,40,46,50^

29 $$ {\text{Z}}_{{{\text{rot}}}} \left( T \right) = \frac{1}{\upsilon }\left\{ {\frac{1}{3} + \frac{T}{{\Theta_{{{\text{rot}}}} }} + \frac{1}{15}\frac{{\Theta_{{{\text{rot}}}} }}{T} + \frac{4}{315}\left( {\frac{{\Theta_{{{\text{rot}}}} }}{T}} \right)^{2} } \right\}, $$

30 $$ {\text{Z}}_{{{\text{tra}}}} \left( T \right) = \left( {\frac{{m{\text{k}}_{{\text{B}}} T}}{{2{\uppi }\hbar^{2} }}} \right)^{{\tfrac{3}{2}}} {\text{V,}} $$

where V is satisfied by pV = R*T*, *m* is the mass of gas molecules enclosed in volume V, the gas pressure is denoted by *p*, R is the molar gas constant, $\Theta_{{{\text{rot}}}} = \hbar^{{2}} /{2}\pi \mu r_{{\text{e}}}^{{2}} {\text{k}}_{{\text{B}}}$ is the characteristic temperature of the gas. The parameter *υ* takes the value 2 if the gas is homonuclear, and 1 for heteronuclear gas molecules. Using the expression for the partition function, explicit equations for molar entropy, enthalpy, Gibbs free energy and constant pressure heat capacity are developed for the DSO as follows.

### Molar entropy equation for the deformed Schiöberg oscillator

The molar entropy (J mol^−1^ K^−1^) of the system can be evaluated from the relation^53^

31 $$ {\text{S}}\left( T \right) = {\text{R}}\ln {\text{Z}} + {\text{R}}T\left( {\frac{\partial }{\partial T}\ln {\text{Z}}} \right)_{{\text{V}}} . $$

Substituting the expression Z (*T*) = Z_vib_Z_rot_Z_tra_ into (31) and using Eqs. (27), (29) and (30) in the result, one obtains

32 $$ {\text{S}}\left( T \right) = \frac{5}{{2}}{\text{R}} + {\text{R}}\left( {\ln {\text{Z}} + \frac{{T{{{\rm Z}^{\prime}}}_{{{\text{vib}}}} }}{{{\text{Z}}_{{{\text{vib}}}} }}} \right) + \frac{{\text{R}}}{\upsilon }\left\{ {\frac{T}{{\Theta_{{{\text{rot}}}} }} - \frac{1}{15}\frac{{\Theta_{{{\text{rot}}}} }}{T} - \frac{8}{315}\left( {\frac{{\Theta_{{{\text{rot}}}} }}{T}} \right)^{2} } \right\} $$

where for compactness, the following abbreviation is used

33 $$ \begin{aligned} T{{{\rm Z}^{\prime}}}_{{{\text{vib}}}} & = \left( {\omega - \lambda_{0}^{2} + \frac{{\lambda_{0} }}{2\varsigma }} \right){\text{A}}_{0} - \left( {\omega - \lambda_{1}^{2} + \frac{{\lambda_{1} }}{2\varsigma }} \right){\text{A}}_{1} - \left( {\omega + \frac{1}{2}} \right){\text{A}}_{2} \\ & \;\;\;\, - \left( {\omega + 4\kappa \varsigma^{2} + \frac{1}{2}} \right){\text{A}}_{3} + \frac{1}{4\varsigma }\left( {\eta_{0} {\text{e}}^{{\eta_{0}^{2} }} - \eta_{1} {\text{e}}^{{\eta_{1}^{2} }} } \right){\text{e}}^{{ - 2\omega - 4\kappa \varsigma^{2} }} . \\ \end{aligned} $$

### Molar enthalpy model of the DSO

The molar enthalpy (J mol^−1^) of the DSO can be deduced from the expression^54^

34 $$ {\text{H}}\left( T \right) = {\text{R}}T^{2} \left( {\frac{\partial }{\partial T}\ln {\text{Z}}} \right)_{{\text{V}}} + {\text{R}}T{\text{V}}\left( {\frac{\partial }{{\partial {\text{V}}}}\ln {\text{Z}}} \right)_{T} . $$

The substitution Z (*T*) = Z_vib_Z_rot_Z_tra_ and Eqs. (27), (29) and (30) and (34) yields

35 $$ {\text{H}}\left( T \right) = \frac{5}{{2}}{\text{R}}T + {\text{R}}T\left( {\frac{{T{{{\rm Z}^{\prime}}}_{{{\text{vib}}}} }}{{{\text{Z}}_{{{\text{vib}}}} }}} \right) + \frac{{{\text{R}}T}}{\upsilon }\left\{ {\frac{T}{{\Theta_{{{\text{rot}}}} }} - \frac{1}{15}\frac{{\Theta_{{{\text{rot}}}} }}{T} - \frac{8}{315}\left( {\frac{{\Theta_{{{\text{rot}}}} }}{T}} \right)^{2} } \right\}. $$

Equation (35) can be used to compute molar enthalpy data for diatomic substances. However, to enable the results obtained in this study to be compared with available literature, scaled values of (35) are needed. The scaled molar enthalpy is written as^44,45^

36 $$ {\text{H}}_{{{\text{scaled}}}} = {\text{ H }}{-}{\text{ h}}_{{{298}.{15}}} , $$

where h_298.15_ is given by (35), it denotes the molar enthalpy of the molecules calculated at temperature of 298.15 K and pressure of 0.1 MPa.

### Molar Gibbs free energy of the DSO

Here, the analytical equation for molar Gibbs free energy is derived for the DSO. The Gibbs free energy is given by

37 $$ {\text{G }} = {\text{ H }}{-}T{\text{S}}. $$

Replacing (34) and (31) into (37) gives

38 $$ {\text{G }} = \, {-}{\text{ lnZ}}_{{{\text{vib}}}} {-}{\text{ lnZ}}_{{{\text{rot}}}} {-}{\text{ lnZ}}_{{{\text{tra}}}} . $$

For the purpose of relating to observed data, the scaled Gibbs free energy is defined as^44,45^

39 $$ {\text{G}}_{{{\text{scaled}}}} = \, {-} \, \left( {{\text{G }}{-}{\text{ H}}_{{{298}.{15}}} } \right)/T. $$

### Isobaric molar specific heat capacity model of the DSO

The constant pressure (isobaric) molar heat capacity (in J mol^−1^ K^−1^) is evaluated from ${\text{C}}_{p} = \tfrac{{\partial \,{\text{H}}}}{\partial \,T}$^34,40^. Substituting expression (35) into this equation gives

40 $$ {\text{C}}_{p} = \frac{5}{{2}}{\text{R}} + {\text{R}}\left\{ {T^{2} \left( {\frac{{{{{\rm Z}^{\prime\prime}}}_{{{\text{vib}}}} }}{{{\text{Z}}_{{{\text{vib}}}} }}} \right) - \left( {T\frac{{{{{\rm Z}^{\prime}}}_{{{\text{vib}}}} }}{{{\text{Z}}_{{{\text{vib}}}} }}} \right)^{2} } \right\} + \frac{{\text{R}}}{\upsilon }\left\{ {\frac{2T}{{\Theta_{{{\text{rot}}}} }} + \frac{8}{315}\left( {\frac{{\Theta_{{{\text{rot}}}} }}{T}} \right)^{2} } \right\}, $$

where ${{{\rm Z}^{\prime}}}_{{{\text{vib}}}}$ and ${{{\rm Z}^{\prime\prime}}}_{{{\text{vib}}}}$ are given by Eqs. (33) and (41), respectively

41 $$ \begin{aligned} T^{2} {{{\rm Z}^{\prime\prime}}}_{{{\text{vib}}}} & = \left\{ {\left( {\lambda_{0}^{2} - \omega + 1} \right)^{2} - \frac{{\lambda_{0} T}}{2\varsigma }\left( {\lambda_{0}^{2} - 2\omega + \frac{1}{2}} \right) - 1} \right\}{\text{A}}_{0} \, - \left\{ {\left( {\lambda_{1}^{2} - \omega + 1} \right)^{2} - \frac{{\lambda_{1} T}}{2\varsigma }\left( {\lambda_{1}^{2} - 2\omega + \frac{1}{2}} \right) - 1} \right\}{\text{A}}_{1} \\ & \;\;\;\, - \left\{ {\left( {\omega + \frac{1}{2}} \right)^{2} - \frac{1}{2}} \right\}{\text{A}}_{2} - \left\{ {\left( {\omega + 4\kappa \varsigma^{2} - \frac{1}{2}} \right)^{2} - \frac{1}{2}} \right\}{\text{A}}_{3} - \frac{T}{4\varsigma }\left( {\eta_{0} {\text{e}}^{{\eta_{0}^{2} }} - \eta_{1} {\text{e}}^{{\eta_{1}^{2} }} } \right){\text{e}}^{{ - \omega - 4\kappa \varsigma^{2} }} . \\ \end{aligned} $$

## Results and discussion

In this section, the equation derived for the energy levels and thermochemical functions are applied to diatomic substances including ^7^Li_2_ (2 ^3^Π_g_), NaBr (X ^1^Σ^+^), KBr (X ^1^Σ^+^) and KRb (B ^1^Π) molecules. The model parameters for these molecules are given in Table 1. The experimental values for *D*_e_, *r*_e_, *ω*_e_ and *α*_e_ were obtained from publications^77–79^. The values of the potential parameters also listed in Table 1 were computed with Eqs. (3) and (6).

**Table 1:** Model parameters of the diatomic molecules investigated in this study.

Diatomic molecule	Molecular state	Spectroscopic parameter				Refs.	Potential parameter	
		*D*_e_ (cm^−1^)	*r*_e_ (Å)	*ω*_e_ (cm^−1^)	*α*_e_ (cm^−1^)		*q*	*α* (Å^−1^)
^7^Li_2_	2^−3^Π_g_	8479.621	3.8463419	188.6858	0.0003010	^77^	− 4.8242	0.3257
NaBr	X^−1^Σ^+^	27,270.21	2.359	293.2	0.000879	^78^	1.5040	0.3233
KBr	X^−1^Σ^+^	22,734.91	2.937	196.6	0.000393	^78^	1.6689	0.2493
KRb	B^−1^Π	4221.0	4.3813	61.256	0.0000741	^79^	− 11.4632	0.3994

To numerically affirm the accuracy of the model equations, the percentage average absolute deviation (PAAD) of the predicted results from the observed data for the molecule is employed as accuracy indicator. The PAAD values are interpreted according to Lippincott condition for the applicability of a model equation. The Lippincott criterion requires that the PAAD value of the predicted data from the observed data is at most 1% of the experimental results. The smaller the PAAD value, better the model equation. The PAAD value is written in compact form as^80^

42 $$ {\text{PAAD}} = \frac{100}{{N_{{\text{p}}} }}\sum\limits_{n\, = \,1}^{{N_{{\text{p}}} }} {\left| {\frac{{{\text{X}} - {\text{Y}}}}{{\text{Z}}}} \right|_{n} } , $$

where *N*_p_ is the count of observed data, X, Y and Z are chosen in relation to the predicted and observed data.

### Numerical results for potential energies

**Figure 1:**
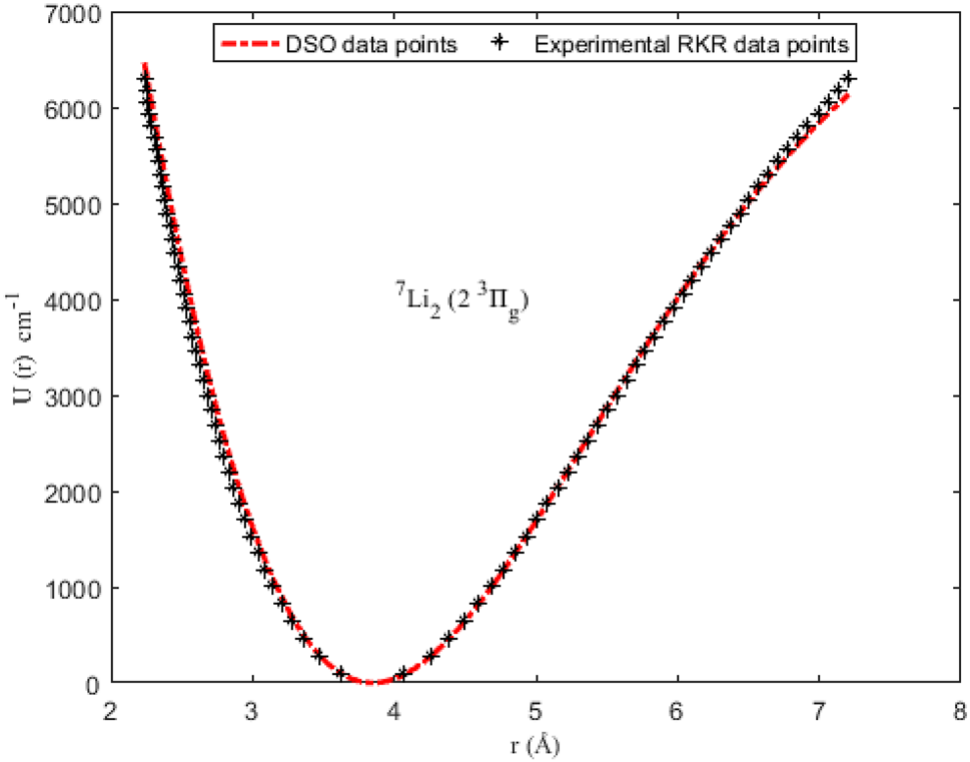
Modeling of deformed Schiöberg potential with experimental RKR interparticle potential energy data for the ^7^Li_2_ (2 ^3^Π_g_) molecule.

**Figure 2:**
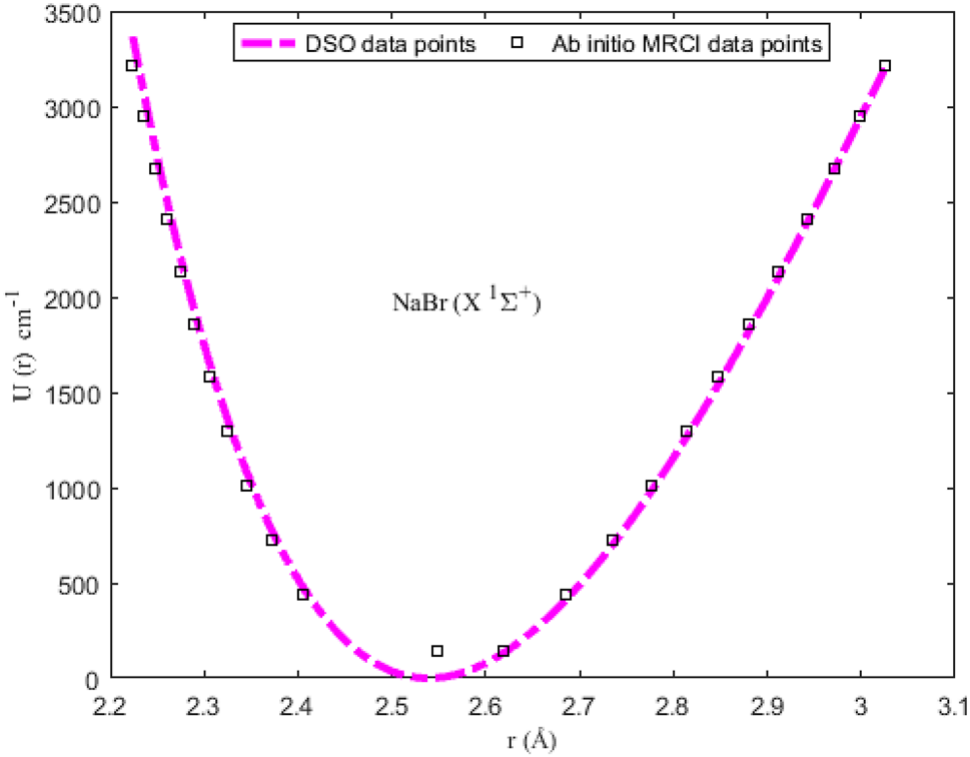
Modeling of deformed Schiöberg potential with ab initio MRCI interparticle potential energy data for the NaBr (X ^1^Σ^+^) molecule.

**Figure 3:**
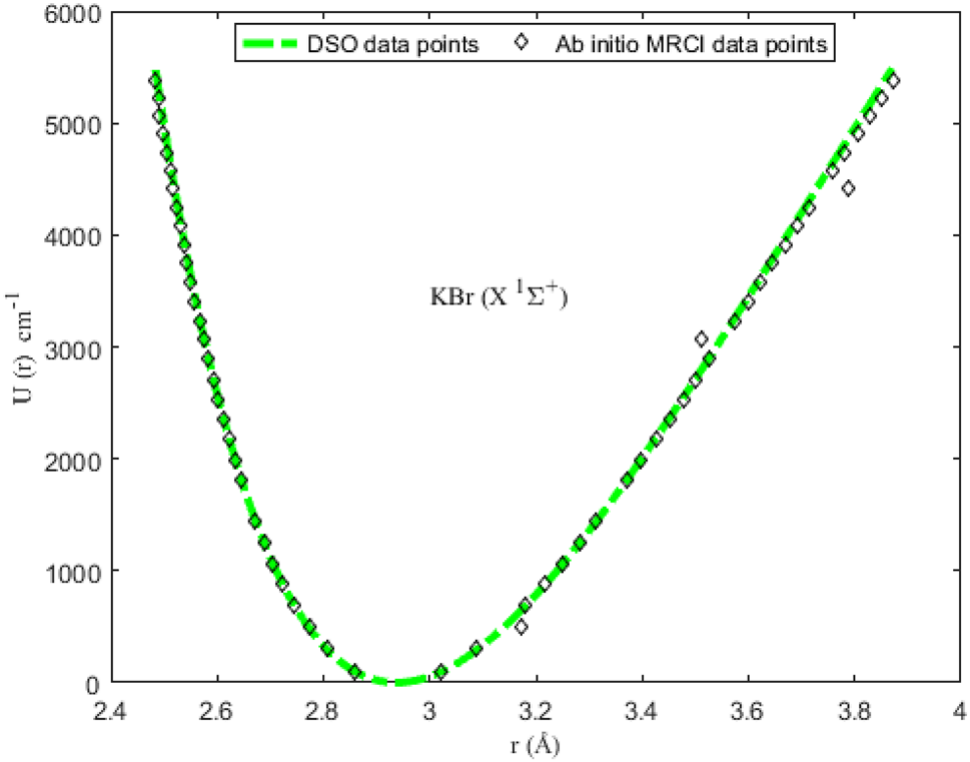
Modeling of deformed Schiöberg potential with ab initio MRCI interparticle potential energy data for the KBr (X ^1^Σ^+^) molecule.

**Figure 4:**
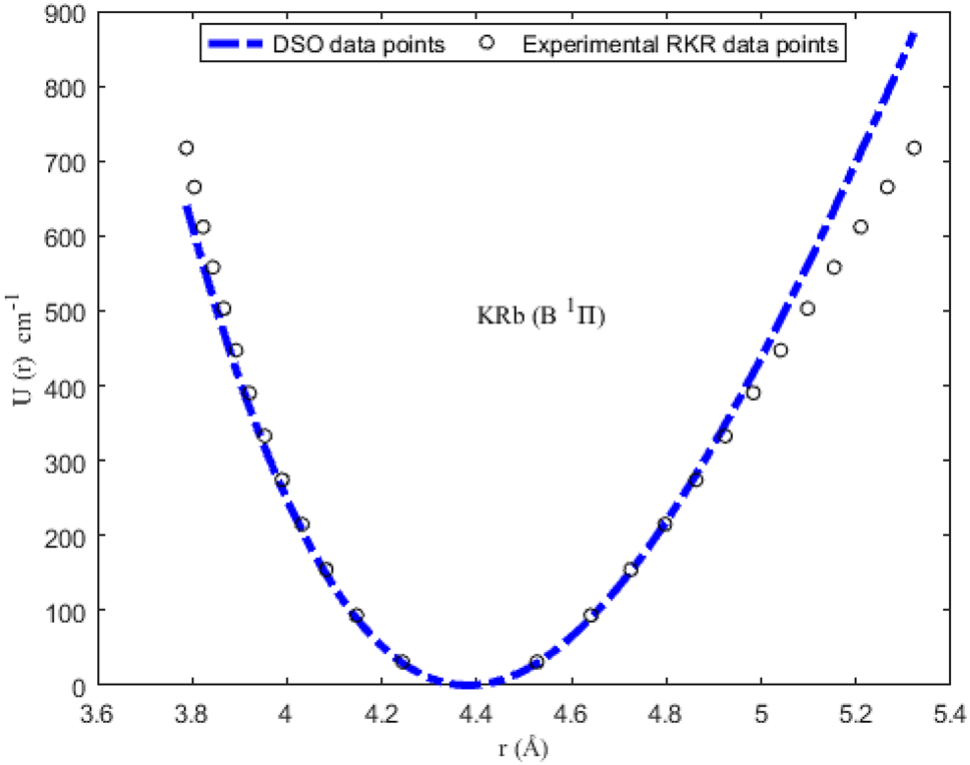
Modeling of deformed Schiöberg potential with experimental RKR interparticle potential energy data for the KRb (B ^1^Π) molecule.

Utilizing the spectroscopic parameters in Table 1, Eq. (1) is used to generate numerical results for the potential energy U (≡ U_min_, U_max_) for different vales of *r* (≡ *r*_min_, *r*_max_). The results obtained are given in Tables 2, 3, 4 and 5. Available experimental Rydberg–Klein–Rees (RKR) data^77,79^, and the multireference configuration interaction (MRCI) data^78^ for the molecules are also included in the tables. The inclusion of the RKR and MRCI data is to allow for comparison of the predicted values of the potential energies with the observed data for the molecules. The variation in potential energy of the molecules as a function of interparticle separation is given in Figs. 1, 2, 3 and 4. The experimental RKR data are also plotted in the figures. The figures show that the computed potential energy of the molecules agree with the experimental data for the molecules.

The accuracy of the DSO to model the experimental RKR data can be determined by letting X = RKR, Y = U and Z = *D*_e_ in Eq. (42). With the help of the resulting expression and the data in Tables 2, 3, 4 and 5, the PAAD values obtained are 1.3319%, 0.2108%, 0.2359% and 0.8841% for the ^7^Li_2_ (2 ^3^Π_g_), NaBr (X ^1^Σ^+^), KBr (X ^1^Σ^+^) and KRb (B ^1^Π) molecules, respectively. Therefore, based on the Lippincott criterion, it can be inferred that the DSO could satisfactorily reproduce the experimental RKR and MRCI results for the selected diatomic molecules.

**Table 2:** Potential energies, bound-state energy eigenvalues and experimental Rydberg–Klein–Rees data for the ^7^Li_2_ (2 ^3^Π_g_) molecule.

*n*	*r* (Å)^77^		U (cm^-1^)		Energy (cm^−1^)	
	*r*_min_	*r*_max_	U_min_	U_max_	RKR^77^	*E*_*n*_
0	3.62168	4.07370	99.3651	89.5434	93.8116	94.0400
1	3.46650	4.25411	295.2991	272.2217	280.1512	281.1230
2	3.36240	4.38449	491.2207	454.4276	464.2410	466.5924
3	3.27941	4.49478	686.7777	635.8801	646.1710	650.4376
4	3.20885	4.59362	881.5848	816.4616	826.0218	832.6477
5	3.14678	4.68493	1075.2478	996.0476	1003.8648	1013.2117
6	3.09100	4.77089	1267.4681	1174.5370	1179.7632	1192.1182
7	3.04013	4.85285	1458.0222	1351.8158	1353.7728	1369.3557
8	2.99133	4.93171	1654.6885	1527.7589	1525.9427	1544.9124
9	2.94974	5.00811	1833.0904	1702.2508	1696.3158	1718.7760
10	2.90905	5.08253	2017.3513	1875.1969	1864.9297	1890.9342
11	2.87084	5.15534	2199.1742	2046.5126	2031.8171	2061.3742
12	2.83479	5.22682	2378.5610	2216.0897	2197.0063	2230.0833
13	2.80066	5.29720	2555.4324	2383.8394	2360.5220	2397.0479
14	2.76827	5.36667	2729.6307	2549.6814	2522.3854	2562.2546
15	2.73744	5.43540	2901.1841	2713.5676	2682.6150	2725.6895
16	2.70803	5.50352	3070.0592	2875.4173	2841.2267	2887.3382
17	2.67993	5.57113	3236.1740	3035.1281	2998.2341	3047.1862
18	2.65302	5.63835	3399.6110	3192.6858	3153.6493	3205.2185
19	2.62723	5.70525	3560.2392	3347.9912	3307.4827	3361.4200
20	2.60246	5.77192	3718.1861	3501.0196	3459.7432	3515.7748
21	2.57865	5.83843	3873.3936	3651.7224	3610.4388	3668.2670
22	2.55574	5.90483	4025.8546	3800.0255	3759.5765	3818.8802
23	2.53366	5.97118	4175.6781	3945.8947	3907.1623	3967.5974
24	2.51238	6.03754	4322.7440	4089.3093	4053.2015	4114.4016
25	2.49183	6.10394	4467.2432	4230.1963	4197.6986	4259.2749
26	2.47199	6.17045	4609.0505	4368.5789	4340.6574	4402.1993
27	2.45280	6.23709	4748.3528	4504.3843	4482.0808	4543.1562
28	2.43424	6.30390	4885.0752	4637.5912	4621.9708	4682.1265
29	2.41627	6.37093	5019.3102	4768.2049	4760.3286	4819.0909
30	2.39886	6.43822	5151.0976	4896.2160	4897.1540	4954.0292
31	2.38198	6.50581	5280.4957	5021.6199	5032.4453	5086.9209
32	2.36559	6.57373	5407.6584	5144.3987	5166.1996	5217.7451
33	2.34968	6.64203	5532.5226	5264.5739	5298.4115	5346.4801
34	2.33421	6.71075	5655.2732	5382.1512	5429.0739	5473.1037
35	2.31916	6.77995	5775.9516	5497.1711	5558.1767	5597.5934
36	2.30449	6.84968	5894.7729	5609.6559	5685.7071	5719.9256
37	2.29019	6.92001	6011.7216	5719.6579	5811.6486	5840.0767
38	2.27622	6.99099	6127.0372	5827.1948	5935.9808	5958.0219
39	2.26255	7.06271	6240.8901	5932.3405	6058.6790	6073.7360
40	2.24915	7.13525	6353.4619	6035.1462	6179.7135	6187.1933
41	2.23599	7.20872	6464.9443	6135.6961	6299.0486	6298.3671

**Table 3:** Potential energies, bound-state energy eigenvalues and experimental Rydberg–Klein–Rees (RKR) data for the NaBr (X ^1^Σ^+^) molecule.

*n*	*r* (Å)^78^		U (cm-^−1^)		Energy (cm^−1^)	
	*r*_min_	*r*_max_	U_min_	U_max_	MRCI^78^	*E*_*n*_
0	2.548	2.619	1.82	132.43	146.31	146.39
1	2.406	2.686	472.01	414.84	436.99	437.42
2	2.373	2.735	766.72	699.12	725.28	726.31
3	2.346	2.776	1073.09	978.32	1011.18	1013.06
4	2.325	2.814	1355.95	1265.55	1294.71	1297.68
5	2.306	2.848	1648.18	1542.47	1575.85	1580.19
6	2.289	2.881	1940.89	1826.77	1854.66	1860.59
7	2.274	2.912	2225.15	2105.94	2131.14	2138.91
8	2.260	2.942	2513.65	2385.76	2405.33	2415.14
9	2.247	2.971	2802.60	2664.10	2677.29	2689.31
10	2.235	2.999	3088.16	2939.15	2947.14	2961.42
11	2.224	3.026	3366.49	3209.44	3215.02	3231.49

**Table 4:** Potential energies, bound-state energy eigenvalues and experimental Rydberg–Klein–Rees (RKR) data for the KBr (X ^1^Σ^+^) molecule.

*n*	*r* (Å)^78^		U (cm^−1^)		Energy (cm^−1^)	
	*r*_min_	*r*_max_	U_min_	U_max_	MRCI^78^	*E*_*n*_
0	2.859	3.021	99.71	96.39	103.06	98.18
1	2.807	3.088	294.60	290.04	298.75	293.48
2	2.773	3.173	488.59	649.16	492.58	487.48
3	2.746	3.178	685.14	673.56	685.27	680.21
4	2.724	3.216	875.81	869.08	876.24	871.65
5	2.705	3.250	1064.28	1057.81	1066.02	1061.83
6	2.688	3.282	1252.86	1245.86	1254.26	1250.75
7	2.672	3.313	1448.60	1436.50	1441.10	1438.41
8	2.645	3.370	1821.87	1805.52	1810.71	1624.83
9	2.633	3.398	2006.21	1994.22	1993.58	1810.01
10	2.622	3.425	2185.72	2180.08	2174.95	1993.96
11	2.611	3.451	2375.72	2362.24	2354.85	2176.69
12	2.601	3.477	2557.89	2547.13	2533.63	2358.20
13	2.592	3.502	2729.79	2727.15	2710.84	2538.51
14	2.582	3.527	2929.93	2909.06	2886.78	2717.61
15	2.574	3.511	3097.19	2792.43	3061.56	2895.52
16	2.566	3.575	3270.99	3262.68	3234.79	3072.25
17	2.558	3.599	3451.52	3441.20	3406.84	3247.80
18	2.551	3.623	3615.15	3620.58	3577.72	3422.17
19	2.543	3.646	3808.81	3793.13	3747.11	3595.39
20	2.537	3.669	3958.83	3966.17	3915.33	3767.44
21	2.530	3.692	4139.15	4139.55	4082.43	3938.34
22	2.523	3.715	4325.31	4313.14	4248.08	4108.10
23	2.517	3.788	4489.64	4864.20	4412.56	4276.73
24	2.511	3.760	4658.46	4652.95	4575.96	4444.22
25	2.505	3.782	4831.86	4818.95	4738.01	4610.59
26	2.499	3.805	5009.96	4992.29	4898.82	4775.84
27	2.491	3.827	5254.88	5157.81	5058.60	4939.99
28	2.489	3.849	5317.47	5322.97	5217.14	5103.03
29	2.484	3.871	5476.37	5487.70	5374.41	5264.97

**Table 5:** Potential energies, bound-state energy eigenvalues and experimental Rydberg–Klein–Rees (RKR) data for the KRb (X ^1^Σ^+^) molecule.

*n*	*r* (Å)^79^		U (cm^−1^)		Energy (cm^−1^)	
	*r*_min_	*r*_max_	U_min_	U_max_	RKR^79^	*E*_*n*_
0	4.2436	4.5271	29.6870	29.8140	31.3150	30.5658
1	4.1469	4.6413	89.0765	90.4331	93.4456	91.4773
2	4.0826	4.7250	147.9232	152.5066	154.6748	152.0427
3	4.0320	4.7969	205.7800	216.1491	215.0026	212.2606
4	3.9897	4.8624	262.2811	281.4223	274.4290	272.1295
5	3.9531	4.9241	317.3394	348.5162	332.9540	331.6479
6	3.9209	4.9834	370.6354	417.5102	390.5776	390.8143
7	3.8925	5.0410	421.4957	488.2304	447.2998	449.6271
8	3.8667	5.0978	470.8877	561.0723	503.1206	508.0847
9	3.8437	5.1539	517.5171	635.6151	558.0400	566.1857
10	3.8230	5.2099	561.6050	712.2045	612.0580	623.9283
11	3.8044	5.2660	602.9538	790.7417	665.1746	681.3110
12	3.7878	5.3224	641.2558	871.1730	717.3898	738.3320

### Applicability of the Pekeris approximation scheme to diatomic systems

**Figure 5:**
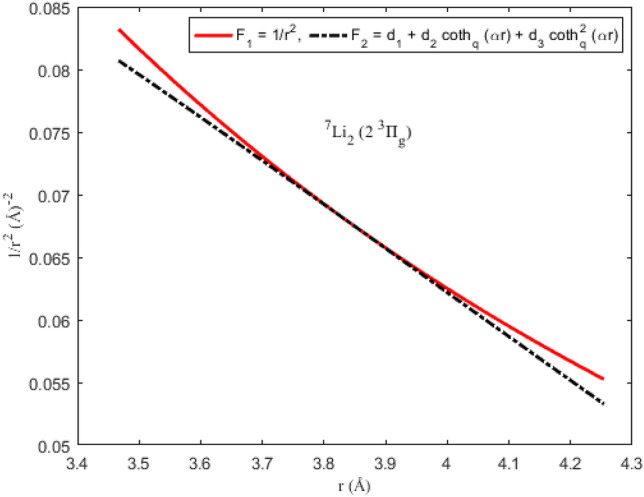
Modeling of the Pekeris approximation scheme F2 to the function F_1_ for the ^7^Li_2_ (2 ^3^Π_g_) molecule.

**Figure 6:**
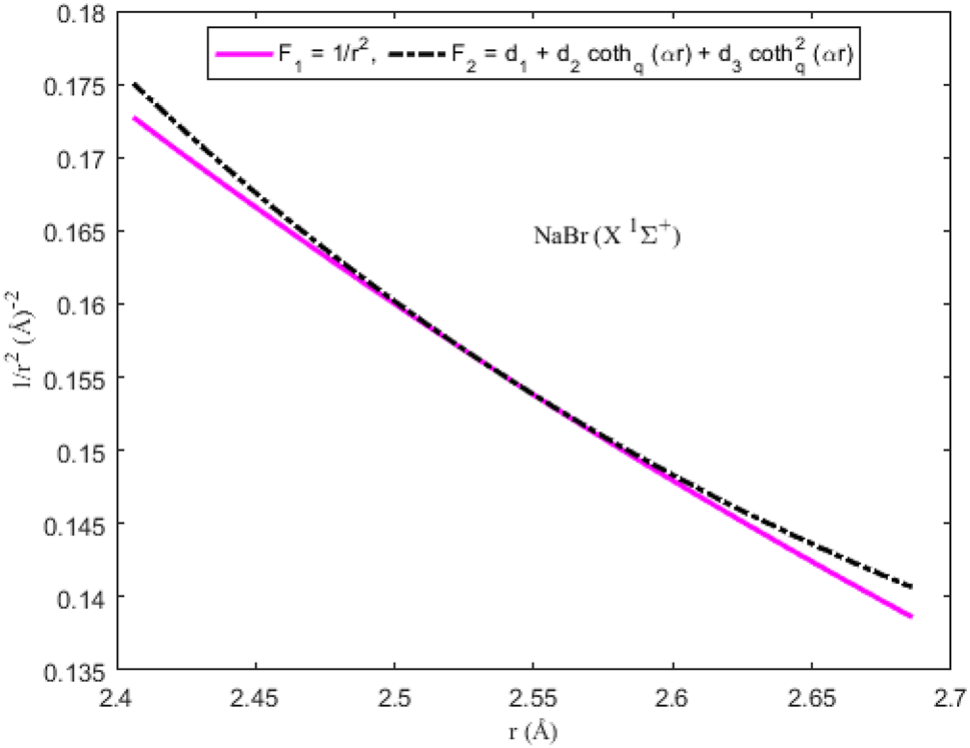
Modeling of the Pekeris approximation scheme F2 to the function F_1_ for the NaBr (X ^1^Σ^+^) molecule.

**Figure 7:**
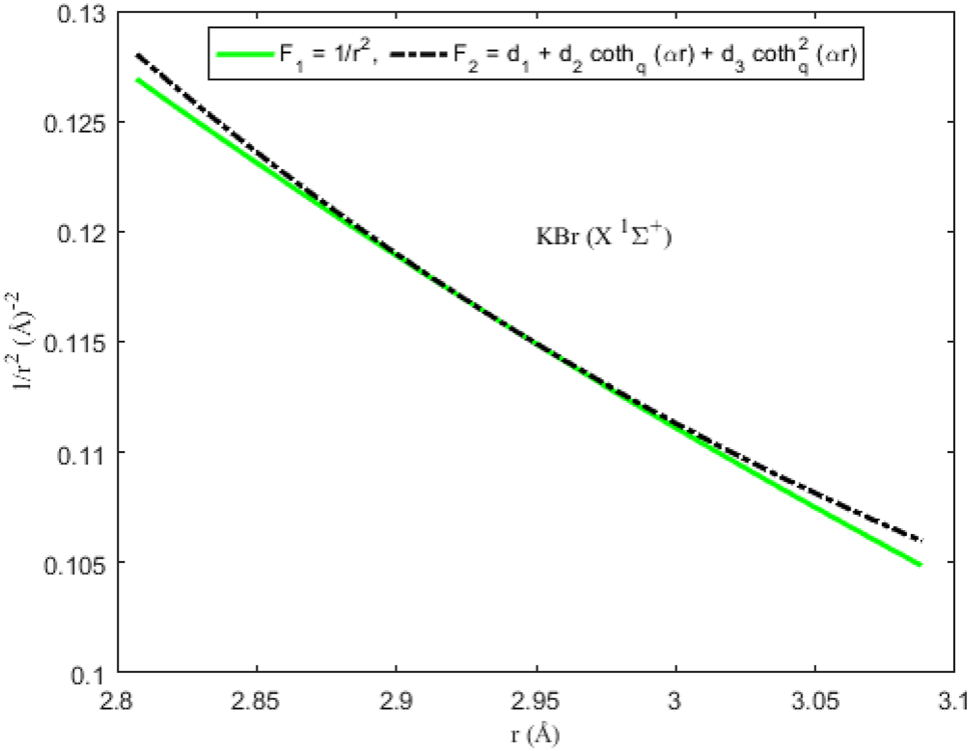
Modeling of the Pekeris approximation scheme F2 to the function F_1_ for the KBr (X ^1^Σ^+^) molecule.

**Figure 8:**
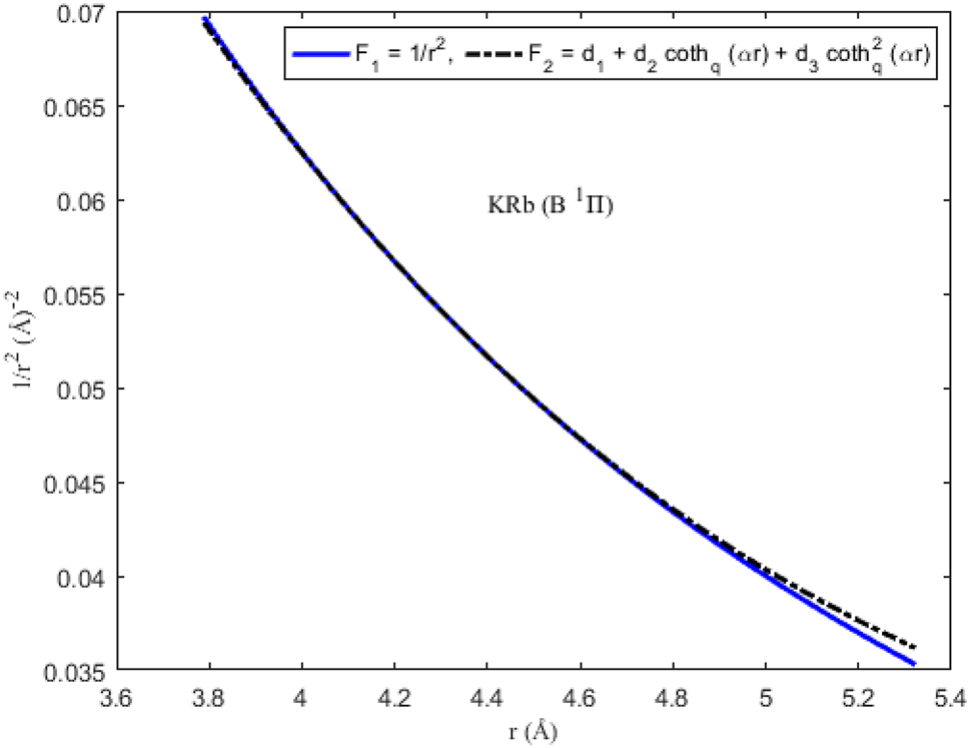
Modeling of the Pekeris approximation scheme F2 to the function F_1_ for the KRb (B ^1^Π) molecule.

To ascertain the significance of the Pekeris-type approximation model (11) suggested for the centrifugal barrier of the SE, the function F_1_ = *r*^−2^ is plotted as a function of interparticle separation. On the same scale and axes, the approximation function F_2_ = *d*_1_ + *d*_2_ coth_*q*_ (*αr*) + *d*_3_ coth_*q*_^2^ (*αr*) is also plotted. The graphical plots depicting F_1_ and F_2_ for the diatomic molecules are shown in Figs. 5, 6, 7 and 8. It is evident from the figures that for the range of *r* chosen for the interparticle separations, the Pekeris approximation F_2_ is a good representation of the observed function F_1_. The implication of the result is that based on the parameters of the diatomic molecules considered in this study, the Pekeris approximation model F_2_ could be used to eliminate the function F_1_ to analytically solve the SE (10).

### Numerical results for pure vibrational state energies

With the aid of Eq. (18), pure vibrational state energies are generated for the selected diatomic molecules. The computed results are summarized in Tables 2, 3, 4 and 5. To quantitatively compare the obtained bound-state energies with the experimental RKR results for the molecules, the parameters in Eq. (42) are adjusted so that X = Z = RKR and Y = *E*_n_. The PAAD values obtained are 1.0956%, 0.2935%, 3.8667% and 1.4629% for the ^7^Li_2_ (2 ^3^Π_g_), NaBr (X ^1^Σ^+^), KBr (X ^1^Σ^+^) and KRb (B ^1^Π) molecules, respectively. Therefore, based on the Lippincott requirement for the applicability of a model equation, the present formula for the pure vibrational state energies could satisfactorily predict the experimental data for the NaBr (X ^1^Σ^+^) molecule, and marginally model the results for ^7^Li_2_ (2 ^3^Π_g_) and KRb (B ^1^Π) molecules. The PAAD value obtained for the KRb (B ^1^Π) molecule is relatively high (≈ 4% of the observed data), suggesting that the present energy levels equation could not satisfactorily predict the observed data for the KRb (B ^1^Π) molecule.

### Investigation of thermochemical properties of diatomic substances

In this section, the thermodynamic functions developed for the DSO are used to analyze the thermochemical properties of pure substances. To substantiate the accuracy of the model equations, numerical data are obtained analytically and the results are compared with the literature on gaseous substances. The experimental results were retrieved from the National Institute of Standards and Technology (NIST) database^81^. The NIST data is available for the gaseous NaBr and KBr molecules only. Thus, our discussions are restricted to these two molecules. PAAD values computed in the temperature range 300–6000 K are used to gauge the accuracy of the model equations.

**Figure 9:**
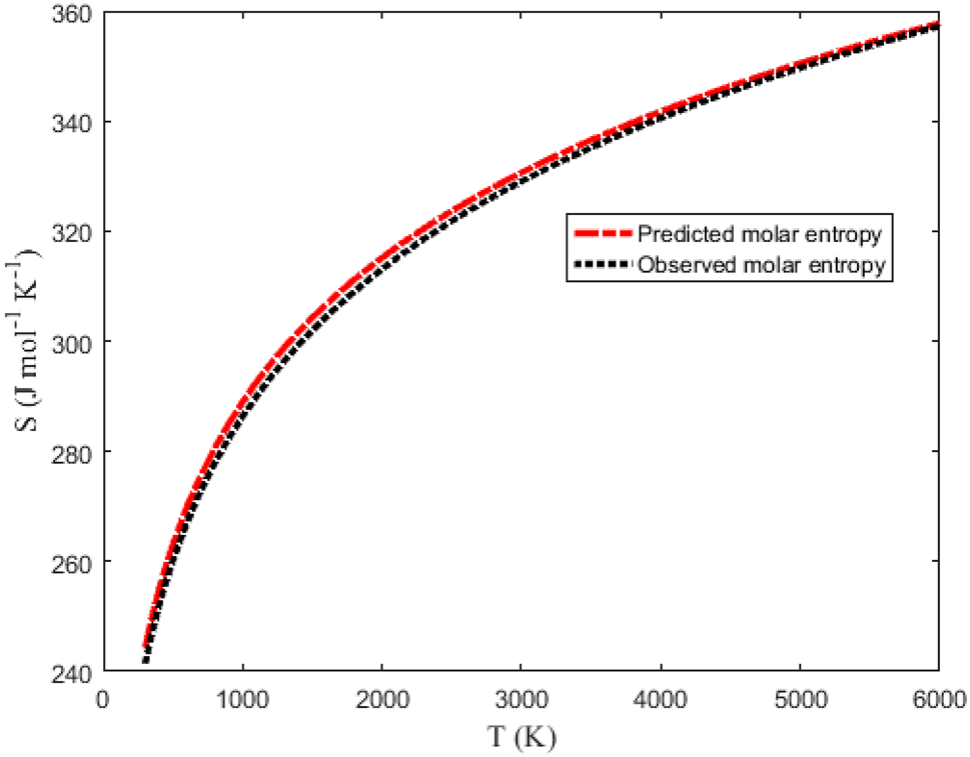
Graphical representation of molar entropy versus temperature for the ground state NaBr molecule.

**Figure 10:**
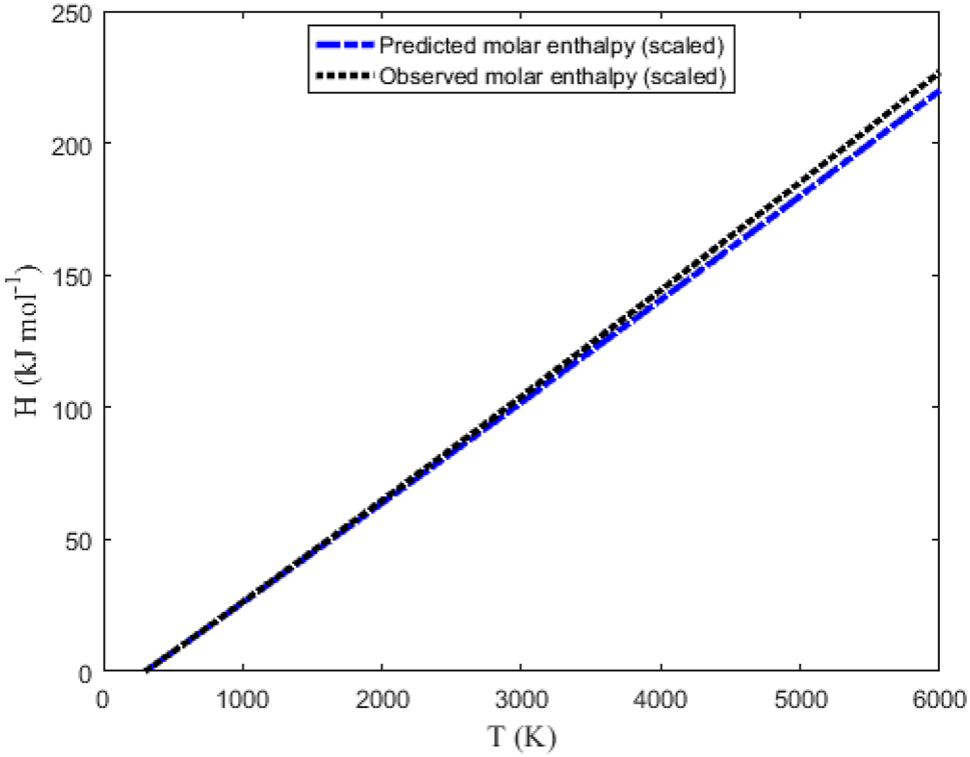
Graphical representation of scaled molar enthalpy versus temperature for the ground state NaBr molecule.

**Figure 11:**
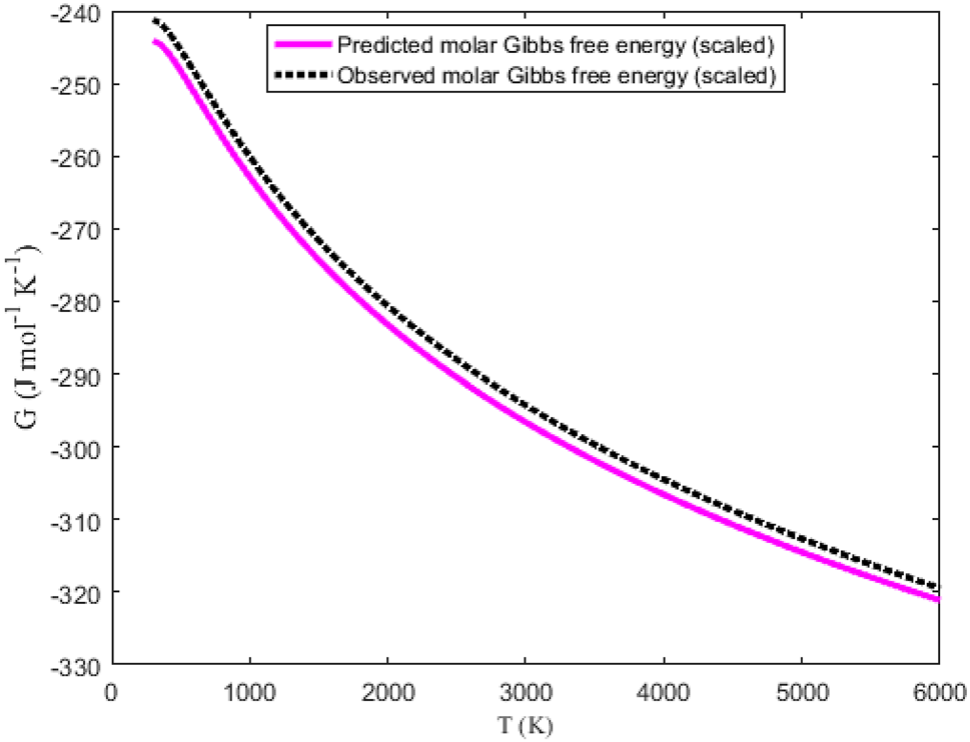
Graphical representation of scaled molar Gibbs free energy versus temperature for the ground state NaBr molecule.

**Figure 12:**
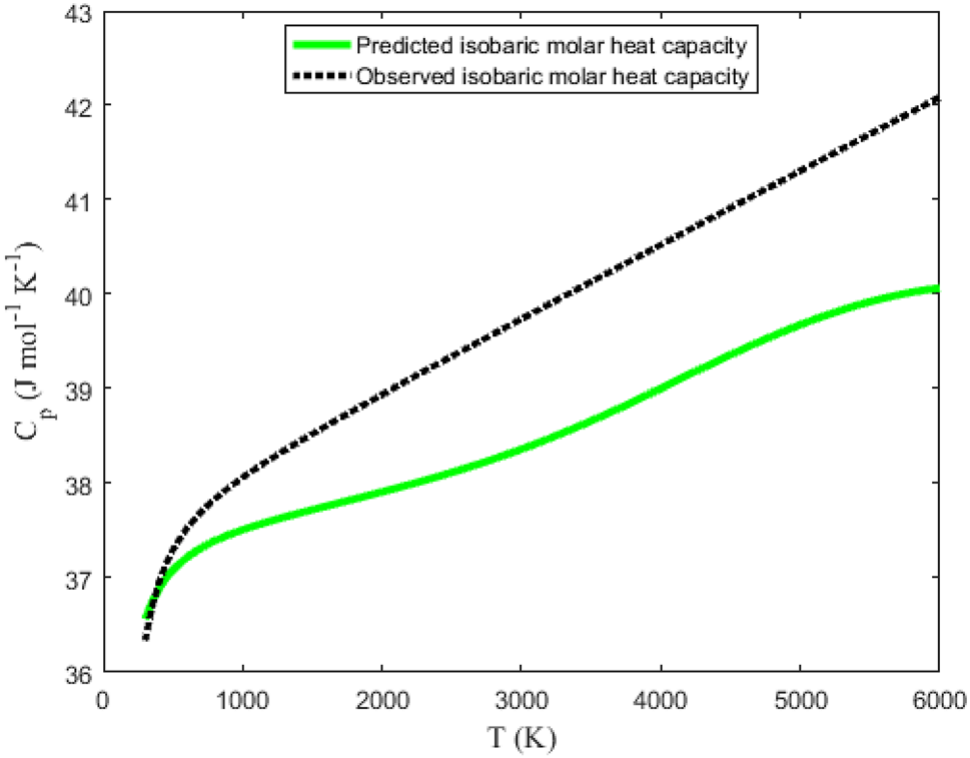
Graphical representation of isobaric molar heat capacity versus temperature for the ground state NaBr molecule.

Tables 6 and 7 summarize the data obtained using Eqs. (32), (36), (39) and (40). The NIST data for the molecules are also listed in the tables under the columns (S_NIST_), (H_NIST_), (G_NIST_) and (C_pNIST_). Graphical plots of the thermochemical equations versus temperature are represented in Figs. 9, 10, 11 and 12. The corresponding NIST data are also plotted in the figures. Due to the similarity in the figures for the NaBr and KBr molecules, only the plots for NaBr molecule are presented.

**Table 6:** Predicted and observed data on molar entropy (J mol^−1^ K^−1^), reduced molar enthalpy (kJ mol^−1^), reduced molar Gibbs free energy (J mol^−1^ K^−1^) and constant pressure molar heat capacity (J mol^−1^ K^−1^) for the NaBr (X ^1^Σ^+^) molecule.

*T* (K)	Entropy		Enthalpy		– RGFE^a^		CPHC^b^		*T* (K)	Entropy		Enthalpy		– RGFE^a^		CPHC^b^	
	S	S_NIST_	H_scaled_	H_NIST_	G_scaled_	G_NIST_	C_p_	C_pNIST_		S	S_NIST_	H_scaled_	H_NIST_	G_scaled_	G_NIST_	C_p_	C_pNIST_
300	241.443	244.325	0.067	0.068	241.219	244.100	36.339	36.562	3100	330.320	331.852	108.160	105.793	295.430	297.725	39.817	38.420
350	247.073	249.976	1.894	1.901	241.663	244.546	36.699	36.748	3200	331.585	333.072	112.146	109.638	296.540	298.810	39.896	38.477
400	251.991	254.893	3.735	3.742	242.653	245.538	36.957	36.889	3300	332.814	334.257	116.139	113.488	297.621	299.867	39.975	38.537
450	256.356	259.244	5.588	5.589	243.937	246.824	37.150	36.999	3400	334.009	335.409	120.141	117.345	298.673	300.895	40.054	38.599
500	260.278	263.147	7.450	7.441	245.379	248.265	37.301	37.087	3500	335.171	336.528	124.150	121.208	299.700	301.897	40.132	38.664
600	267.100	269.921	11.192	11.157	248.447	251.326	37.529	37.220	3600	336.303	337.619	128.167	125.078	300.701	302.875	40.211	38.729
700	272.899	275.666	14.954	14.884	251.536	254.403	37.699	37.317	3700	337.405	338.681	132.192	128.954	301.678	303.828	40.289	38.797
800	277.942	280.654	18.731	18.620	254.529	257.380	37.837	37.394	3800	338.481	339.716	136.225	132.837	302.632	304.759	40.368	38.866
900	282.405	285.062	22.520	22.362	257.383	260.216	37.957	37.456	3900	339.530	340.727	140.265	136.728	303.565	305.668	40.446	38.936
1000	286.410	289.012	26.322	26.111	260.089	262.901	38.065	37.510	4000	340.555	341.713	144.314	140.625	304.477	306.557	40.524	39.006
1100	290.043	292.589	30.133	29.864	262.649	265.440	38.165	37.558	4100	341.557	342.677	148.370	144.529	305.369	307.426	40.603	39.077
1200	293.368	295.859	33.954	33.622	265.073	267.841	38.260	37.603	4200	342.536	343.620	152.434	148.440	306.243	308.277	40.681	39.148
1300	296.434	298.871	37.785	37.385	267.369	270.113	38.351	37.644	4300	343.495	344.542	156.506	152.358	307.098	309.110	40.759	39.219
1400	299.279	301.662	41.625	41.151	269.547	272.268	38.440	37.684	4400	344.433	345.444	160.586	156.284	307.936	309.925	40.837	39.290
1500	301.934	304.263	45.473	44.921	271.619	274.315	38.526	37.722	4500	345.351	346.328	164.674	160.216	308.757	310.724	40.916	39.359
1600	304.423	306.699	49.330	48.695	273.592	276.264	38.611	37.760	4600	346.251	347.194	168.769	164.156	309.562	311.508	40.994	39.426
1700	306.767	308.989	53.195	52.473	275.476	278.122	38.695	37.798	4700	347.134	348.042	172.873	168.102	310.352	312.276	41.072	39.493
1800	308.981	311.151	57.069	56.255	277.276	279.898	38.778	37.835	4800	347.999	348.875	176.984	172.054	311.128	313.030	41.150	39.557
1900	311.080	313.197	60.950	60.040	279.000	281.597	38.860	37.873	4900	348.849	349.691	181.103	176.013	311.889	313.770	41.228	39.618
2000	313.075	315.141	64.840	63.830	280.655	283.226	38.941	37.911	5000	349.682	350.492	185.230	179.978	312.636	314.496	41.307	39.677
2100	314.977	316.991	68.739	67.623	282.244	284.790	39.022	37.950	5100	350.501	351.278	189.364	183.948	313.371	315.210	41.385	39.733
2200	316.794	318.758	72.645	71.420	283.774	286.294	39.103	37.990	5200	351.305	352.050	193.506	187.924	314.093	315.911	41.463	39.785
2300	318.534	320.447	76.559	75.221	285.247	287.743	39.183	38.031	5300	352.096	352.808	197.657	191.905	314.802	316.600	41.541	39.834
2400	320.203	322.067	80.481	79.026	286.669	289.139	39.263	38.074	5400	352.873	353.553	201.815	195.891	315.500	317.277	41.619	39.880
2500	321.808	323.622	84.412	82.836	288.043	290.488	39.342	38.117	5500	353.638	354.286	205.981	199.881	316.187	317.944	41.697	39.921
2600	323.352	325.118	88.350	86.650	289.371	291.791	39.422	38.163	5600	354.390	355.005	210.154	203.875	316.862	318.599	41.775	39.959
2700	324.841	326.559	92.296	90.468	290.658	293.052	39.501	38.210	5700	355.130	355.713	214.336	207.873	317.527	319.244	41.853	39.992
2800	326.279	327.950	96.250	94.292	291.904	294.274	39.580	38.259	5800	355.858	356.409	218.525	211.873	318.182	319.879	41.931	40.021
2900	327.670	329.293	100.212	98.120	293.114	295.459	39.660	38.310	5900	356.576	357.093	222.722	215.877	318.826	320.504	42.010	40.045
3000	329.016	330.593	104.182	101.954	294.288	296.608	39.739	38.364	6000	357.282	357.766	226.927	219.882	319.461	321.119	42.088	40.066

RGFE^a^: Reduced Gibbs free energy; CPHC^b^: Constant pressure heat capacity.

**Table 7:** Predicted and observed data on molar entropy (J mol^−1^ K^−1^), reduced molar enthalpy (kJ mol^−1^), reduced molar Gibbs free energy (J mol^−1^ K^−1^) and constant pressure molar heat capacity (J mol^−1^ K^−1^) for the KBr (X ^1^Σ^+^) molecule.

*T* (K)	Entropy		Enthalpy		– RGFE^a^		CPHC^b^		*T* (K)	Entropy		Enthalpy		– RGFE^a^		CPHC^b^	
	S	S_NIST_	H_scaled_	H_NIST_	G_scaled_	G_NIST_	C_p_	C_pNIST_		S	S_NIST_	H_scaled_	H_NIST_	G_scaled_	G_NIST_	C_p_	C_pNIST_
300	250.761	255.952	0.068	0.069	250.533	255.723	36.932	37.076	3100	339.981	344.027	108.383	106.407	305.018	309.702	39.853	38.816
350	256.471	261.675	1.921	1.925	250.983	256.175	37.155	37.180	3200	341.247	345.261	112.372	110.293	306.131	310.794	39.932	38.898
400	261.444	266.645	3.783	3.786	251.987	257.180	37.316	37.258	3300	342.477	346.459	116.369	114.187	307.214	311.857	40.012	38.982
450	265.846	271.037	5.652	5.651	253.287	258.480	37.440	37.319	3400	343.673	347.624	120.374	118.090	308.269	312.892	40.091	39.067
500	269.796	274.972	7.526	7.518	254.744	259.936	37.541	37.368	3500	344.836	348.757	124.387	122.001	309.297	313.900	40.170	39.152
600	276.656	281.792	11.289	11.259	257.841	263.028	37.701	37.447	3600	345.969	349.862	128.408	125.920	310.300	314.884	40.249	39.237
700	282.477	287.569	15.066	15.007	260.955	266.131	37.829	37.508	3700	347.073	350.938	132.437	129.848	311.279	315.844	40.328	39.320
800	287.536	292.581	18.854	18.760	263.968	269.131	37.940	37.561	3800	348.149	351.987	136.474	133.784	312.235	316.781	40.408	39.402
900	292.011	297.008	22.653	22.519	266.840	271.988	38.041	37.607	3900	349.200	353.012	140.519	137.728	313.169	317.697	40.487	39.482
1000	296.024	300.973	26.462	26.281	269.562	274.691	38.136	37.651	4000	350.226	354.013	144.571	141.680	314.083	318.593	40.566	39.558
1100	299.663	304.563	30.280	30.049	272.135	277.246	38.226	37.694	4100	351.229	354.990	148.632	145.640	314.977	319.468	40.645	39.632
1200	302.992	307.845	34.107	33.820	274.570	279.661	38.314	37.735	4200	352.209	355.946	152.700	149.606	315.852	320.326	40.724	39.701
1300	306.063	310.867	37.943	37.596	276.876	281.947	38.400	37.776	4300	353.168	356.881	156.777	153.580	316.708	321.165	40.803	39.766
1400	308.911	313.668	41.787	41.375	279.063	284.114	38.484	37.817	4400	354.107	357.796	160.861	157.559	317.548	321.987	40.882	39.826
1500	311.569	316.278	45.640	45.159	281.143	286.172	38.568	37.859	4500	355.027	358.692	164.953	161.545	318.370	322.793	40.961	39.881
1600	314.061	318.723	49.501	48.947	283.123	288.131	38.650	37.902	4600	355.928	359.569	169.053	165.535	319.177	323.583	41.040	39.930
1700	316.407	321.022	53.370	52.740	285.013	289.999	38.732	37.946	4700	356.811	360.428	173.161	169.530	319.969	324.358	41.119	39.973
1800	318.623	323.193	57.247	56.536	286.819	291.783	38.814	37.991	4800	357.678	361.270	177.277	173.530	320.745	325.118	41.198	40.010
1900	320.724	325.248	61.132	60.338	288.549	293.491	38.895	38.038	4900	358.528	362.095	181.401	177.532	321.508	325.864	41.277	40.041
2000	322.721	327.200	65.026	64.144	290.208	295.128	38.975	38.087	5000	359.363	362.904	185.532	181.538	322.256	326.597	41.356	40.065
2100	324.624	329.060	68.927	67.955	291.802	296.700	39.056	38.139	5100	360.183	363.698	189.672	185.545	322.992	327.317	41.435	40.084
2200	326.443	330.835	72.837	71.772	293.335	298.212	39.136	38.193	5200	360.988	364.476	193.819	189.554	323.715	328.024	41.514	40.095
2300	328.184	332.534	76.755	75.594	294.813	299.667	39.216	38.250	5300	361.779	365.240	197.975	193.564	324.426	328.719	41.593	40.100
2400	329.855	334.163	80.680	79.422	296.238	301.071	39.296	38.310	5400	362.558	365.990	202.138	197.574	325.125	329.402	41.672	40.099
2500	331.461	335.728	84.614	83.256	297.615	302.426	39.376	38.373	5500	363.323	366.726	206.309	201.584	325.812	330.074	41.751	40.091
2600	333.007	337.235	88.556	87.097	298.947	303.736	39.456	38.439	5600	364.076	367.448	210.488	205.592	326.489	330.735	41.830	40.078
2700	334.497	338.687	92.505	90.944	300.236	305.004	39.535	38.508	5700	364.817	368.157	214.675	209.599	327.155	331.385	41.909	40.058
2800	335.937	340.088	96.463	94.798	301.486	306.232	39.615	38.581	5800	365.547	368.853	218.870	213.603	327.810	332.025	41.988	40.032
2900	337.328	341.444	100.428	98.660	302.698	307.423	39.694	38.657	5900	366.265	369.537	223.073	217.605	328.456	332.655	42.067	40.000
3000	338.675	342.756	104.401	102.530	303.875	308.579	39.774	38.735	6000	366.973	370.209	227.283	221.603	329.092	333.276	42.146	39.963

RGFE^a^: Reduced Gibbs free energy; CPHC^b^: Constant pressure heat capacity.

Figure 9 shows the graphical representation of the molar entropy against temperature. The figure shows that the predicted molar entropy agrees with the experimental results. To appraise the quality of the molar entropy model, the parameters X, Y, Z in Eq. (42) are chosen such that X = Z = S_NIST_ and Z = S. The PAAD values deduced are 0.5401% and 1.2919%, for NaBr and KBr molecules, respectively. The obtained PAAD values are within the Lippincott error limit. This means that molar entropy equation proposed for the DSO could satisfactorily predict the NIST data for the gaseous NaBr and KBr molecules.

In the plot shown in Fig. 10, scaled molar enthalpy is plotted as a function of temperature. The agreement between the observed and predicted data is evident in the figure. An estimate of the efficiency of the molar enthalpy model can be obtained by letting X = Z = H_NIST_ and Z = H_scaled_ in Eq. (42). Using the data in Tables 6 and 7, the computed PAAD values are 1.9428% and 1.5639% for the NaBr and KBr molecules, respectively. The PAAD values reveal that the DSO model for the scaled molar enthalpy could marginally predict the experimental results for the gaseous molecules. It is also noted from the tables that as the molecules are excited from moderate to high temperature region, the discrepancy between the predicted and observed data increases. The increased difference could be linked to lowest order approximation used to obtain expression (36). The absence of the quantum correction terms in the molar entropy equation is responsible for PAAD values exceeding 1%.

The variations in molar Gibbs free energy with temperature is graphically represented in Fig. 11. The figure show that the results obtained by analytical computations are in good agreement with the data reported in the NIST database for the gaseous substances. With the help of the data in Tables 6 and 7, the PAAD values obtained are 0.8164% and 1.5957% for the ground state NaBr and KBr molecules, respectively. The obtained PAAD values are deduced by setting X = Z = G_NIST_, Y = G_scaled_ in (42). Based on the Lippincott condition, it can be inferred that the molar Gibbs free energy model for the DSO could satisfactorily predict the Gibbs free energy of the selected diatomic molecules.

In Fig. 12, the constant pressure molar heat capacity is plotted against the temperature of the molecules. From the figure, it is clear that in the low temperature range, the predicted isobaric molar heat capacity agrees with the experimental data for the molecules. However, in the moderate to high temperature domain, the predicted heat capacity results are smaller, and deviate significantly from the observed data. The reason for the relatively high deviation could be associated with the quantum corrections terms which are absent in Eq. (40).

Taking X = Z = C_pNIST_, and Y = C_p_, the PAAD values deduced for the molecules are 2.9770% and 2.4041% for the ground state NaBr and KBr, respectively. The results clearly suggest that the isobaric molar heat capacity could not accurately predict the experimental results for the NaBr and KBr molecules. Nevertheless, the results in the tables suggest that the model could be used to obtain the molar heat capacity of the molecules within the low temperature range.

## Conclusions

In this work, the necessary conditions for a diatomic molecule oscillator were used to construct an improved version of the deformed Schiöberg oscillator (DSO). By employing the parametric Nikiforov-Uvarov solution recipe to solve the radial SE with the DSO, analytical expressions for energy spectra and canonical partition function were obtained. Using the obtained partition function, thermodynamic properties such as molar entropy, enthalpy, Gibbs free energy and isobaric heat capacity were developed for the DSO. The obtained equations were used to analyze the physical properties of diatomic substances including ^7^Li_2_ (2 ^3^Π_g_), NaBr (X ^1^Σ^+^), KBr (X ^1^Σ^+^) and KRb (B ^1^Π) molecules. The percentage average absolute deviation (PAAD) of the predicted data from the experimental data of the molecules is used as the goodness-of-fit indicator. The PAAD values obtained with the DSO are 1.3319%, 0.2108%, 0.2359% and 0.8841% for the molecules. The equation of bound state energy levels gave PAAD of 1.0956%, 0.2935%, 3.8667% and 1.4629% from the experimental data of the ^7^Li_2_ (2 ^3^Π_g_), NaBr (X ^1^Σ^+^), KBr (X ^1^Σ^+^) and KRb (B ^1^Π) molecules. PAAD values were also obtained using the expression for molar entropy, scaled molar enthalpy, scaled molar Gibbs free energy and constant pressure molar heat capacity models. The results obtained for NaBr (X ^1^Σ^+^) molecule are 0.5401%, 1.9428%, 0.8164% and 2.9770%. The corresponding results for KBr (X ^1^Σ^+^) are 1.2919%, 1.5639%, 1.5597% and 2.4041% from the NIST data. The results obtained are in good agreement with theoretic data reported in existing literature and available experimental data on diatomic systems. The results obtained in this study could have practical applications in the many fields of physics and engineering such as solid-state physics, chemical physics, chemical engineering and molecular physics.

## Data Availability

All the data used in this work are in the manuscript.
